# c-Met and miRs in Cancer

**DOI:** 10.3390/biomedicines3010032

**Published:** 2015-01-05

**Authors:** Simona Giglio, Andrea Vecchione

**Affiliations:** Department of Clinical and Molecular Medicine, Sapienza University of Rome, Rome 00161, Italy

**Keywords:** microRNA, c-MET, HGF, cancer

## Abstract

c-Met, a member of the receptor tyrosine kinase family, is involved in a wide range of cellular processes, including tumor survival, cell growth, angiogenesis and metastasis, and resulting in overexpression in many human cancers, leading to a constitutive activation of the downstream pathways. Recently identified MicroRNAs are a family of small noncoding RNA molecules, extensively studied in cancer, that exert their action by inhibiting gene expression at the posttranscriptional level in several biological processes. Aberrant regulation of microRNAs expression has been implicated in the pathogenesis of different human neoplasia. Several publications point out the connections between c-Met and its ligand hepatocyte growth factor (HGF) and microRNAs. This review summarizes the current knowledge about the interplay between c-Met/HGF and microRNAs and provides evidence that microRNAs are a novel and additional system to regulate c-Met expression in tumors. In the future, microRNAs connected to c-Met may provide an additional option to inhibiting this oncogene from orchestrating an invasive growth program.

## 1. The HGF/c-Met Pathway

The c-Met proto-oncogene was originally identified as a fusion gene (tpr-c-Met) in a chemically transformed human osteosarcoma cell line [1]. The gene for c-Met is located on chromosome 7q21–q31 and encodes for a single precursor that is posttranscriptionally processed, giving rise to a 50 kDa extracellular α-chain and a transmembrane 140 kDa β-chain, which are linked by disulfide bonds [2]. c-Met’s ligand has been identified as the hepatocyte growth factor (HGF) which is secreted by fibroblasts and smooth muscle cells [3,4]. Ligand-induced c-Met dimerization leads to phosphorylation of specific tyrosine residues (Tyr1230, Tyr1234 and Tyr1235) in the kinase domain, which, in turn, induces autophosphorylation of the carboxy-terminal bidentate substrate-binding site (Tyr1349 and Tyr1356) of c-Met [5].

Cytoplasmic signaling cascades, mainly mediated by PI3K–AKT and the ERK pathways, modulate cell survival and trigger changes to the plasma membrane, control cell migration and cell adhesion through downstream molecules that include cadherins, integrins, focal adhesion kinase and paxillin pathways [5,6].

Thus, the HGF/c-Met activated signaling promotes a complex biological program named “invasive growth” that results in stimulation of cell motility, invasion, and protection from apoptosis.

The c-Met oncogene is deregulated in different human tumors at multiple levels, mainly through transcriptional deregulation, gene amplification, inadequate degradation, receptor crosstalk or synergies in the downstream signaling.

Recently identified microRNA (miRs) are noncoding small RNA (20–22 nucleotides) that have the potential to regulate at least 20%–30% of all human transcripts and are therefore involved in almost all basic signaling pathways. The regulation of miRNAs biogenesis and function has been extensively reviewed elsewhere [7,8]. Dysregulated miRs contribute to a variety of pathological events, including cancer. Thus, miRs are able to inhibit the expression of major tumor-related genes in carcinogenesis, acting themselves as oncogenes or oncosuppressors [9,10].

Recent evidences show that alteration of miR expression was observed in different human tumor types, and their key role in cancer pathogenesis and response to therapy has been proven [11,12].

In recent years, a rapidly growing number of subsequent papers refined the knowledge about the interplay between c-Met/HGF and miRNAs. In this review, functional studies on regulation of c-Met and miRNAs will be summarized in detail.

## 2. MicroRNAs Regulating c-Met

### 2.1. miR-34 Family Members

The miR-34 family, consist of miR-34a, miR-34b and miR-34c that are frequently silenced in a variety of tumors, indicating their role in tumorigenesis. These three miR-34s are produced from two transcriptional units. MiR-34a is transcribed from chromosome 1, a locus deleted in neuroblastoma, breast, thyroid, and cervical cancer [13,14,15,16], while miR-34b and miR-34c are co-transcribed from a region on chromosome 11.

Several studies pointed out miR-34 as one of the main miR-regulating c-Met.

Indeed He and colleagues demonstrated for the first time the direct interaction between miR-34 and c-Met in mouse embryonic fibroblasts (MEF) cells [17]. Hereafter, c-Met was established as a *bona fide* miR-34 target in different tumors such as melanoma, lung, colon, breast and gastric cancer cells [18]. Particularly in glioblastoma and ovarian cancer, miR-34 family members’ a–b–c expression was inversely correlated with c-Met expression [19,20,21].

Moreover, it was reported by the same authors that miR-34 inhibits cell invasion, proliferation and tumorigenesis, whereas c-Met over-expression partially reversed the cell death and cell cycle arrest induced by miR-34 in brain tumors and glioma [20,21]. Importantly, miR-34 blocked the phosphorylation signal cascade of c-Met, Akt, ERK and compromised c-Met-driven invasion [18].

In a study conducted by Dang *et al.*, it has been demonstrated that the enforced expression of miR-34a mimic enhanced the effect of cell proliferation inhibition and caspase activity induction of agents targeting c-Met in hepatocellular carcinoma [22].

Cai and colleagues identified an inverse relationship between the expression of miR-34c and c-Met, in 10-paired fresh samples from tumor tissues and adjacent normal tissues of laryngeal carcinoma, showing that down-regulated miR-34c is a critical factor that contributes to malignancy in human laryngeal carcinoma by targeting of c-Met [23].

Zhou and collegues demonstrated additional evidence of the pivotal role played by miR-34a in HGF/MET signaling. They found that miR-34 was able to overcome HGF-induced gefitinib resistance in HCC827 and PC-9 cells by modulating c-Met and downstream pathway molecules, suggesting a new strategy for reversing HGF-induced resistance to gefitinib in lung cancers [24].

### 2.2. miR-199a-3p

The miR-199–miR-214 cluster is of particular interest because it is downregulated in the majority of hepatocellular carcinomas (HCCs) [25,26], bladder [27], ovarian [28], and renal carcinomas [29] and in cancer-derived cell lines in experimental neoplastic and preneoplastic conditions [30].

Both Kim and Migliore identified the *c-Met* proto-oncogene as a target of the miR-199a-3p [18,31]. Particularly, they demonstrated that miR-199a-3p inhibits not only proliferation, but also motility and invasive capabilities of tumor cells by downregulating both c-Met and its downstream effector ERK2 [31].

It was recently reported that serum concentration of HGF, the c-Met receptor ligand, was significantly elevated in renal cell carcinoma (RCC) patients compared to healthy individuals, thereby suggesting that miR-199a-3p impairs HGF/c-Met signaling pathway, including STAT3, mTOR and ERK1/2, which is crucial for RCC development, thus also suggesting that this miR may serve as a potential target for RCC therapy [29].

Minna and colleagues reported downregulation of miR-199a-3p in papillary thyroid cancer (PTC) specimens and cell lines, and demonstrated that its restoration in PTC cells reduces c-Met and mTOR protein levels, impairing migration and proliferation and, more interestingly, inducing lethality through an unusual form of cell death similar to methuosis, caused by macropinocytosis dysregulation, unveiling interesting networks including HGF and macropinocytosis pathways [32].

### 2.3. miR-340

miR-340 is downregulated in aggressive breast cancer cell lines and breast cancer tissue specimens, indicating its tumor suppression role. Wu and colleagues demonstrated that miR-340 inhibits c-Met and consequently MMP-2 and MMP-9 expressions by direct targeting of the *c-Met* gene [33].

Another research group reported that low expression of miR-340 is associated with poor prognosis in colorectal cancer and demonstrated that pre-miR-340 administration inhibited growth of colon cancer cells and suppressed c-Met expression *in vitro*. Interestingly, they observed that the colorectal cancer patients with low miR-340 and high c-Met expression had the worst prognosis [34,35].

### 2.4. miR-148a

MiR-148a downregulation is reported in multiple malignancies by different authors, and, its over-expression inhibits growth of pancreatic and prostate cancer cells, promotes apoptosis of colorectal cancer, suppresses angiogenesis of breast cancer and represses metastatic potential of gastric cancer-derived cell lines [36,37,38,39,40].

Zhang and colleagues proved that miR-148a directly target c-Met and abrogate c-Met/Snail signaling in hepatoma cells, providing novel mechanistic insights into the role of miR-148a in ephitelial mesenchymal transition (EMT) and metastasis [41].

Another study confirmed a significant down-regulation of miR-148a in HCC, indicating that this miR exerted its tumor-suppressive effect by regulating the c-Met oncogene, regardless of the DNMT1, the DNA methyltransferase 1, expression level [42,43].

### 2.5. miR-1

Several papers showed the direct interaction between miR-1 and c-Met.

Nasser and colleagues have published the first evidence of the direct binding between miR-1 and the 3' UTR of c-Met, reporting that exogenous miR-1 significantly reduced its expression, thereby reducing cell migration and motility of A549 cells in a c-Met-mediated manner [44]. A significantly lower level of miR-1 compared to the higher level of c-Met expression was observed in aggressive PTC, in chordoma tissues and human primary lung cancer tissues and cell lines [44,45,46].

Recently, Duan and colleagues showed that miR-1 was downregulated in 93.7% of chordoma tissues and its expression was inversely correlated with c-Met expression, indicating that suppressed miR-1 expression in chordoma may in part be a driver for tumor growth, and that miR-1 has the potential to serve as a prognostic biomarker and therapeutic target for chordoma patients [46].

Novello and colleagues demonstrated that the ectopic expression of miR-1 in the U2-OS osteosarcoma cell lines, significantly reduced cell proliferation and cell invasiveness correlated with c-Met down-regulation. They performed a miR profiling in osteosarcoma clinical samples and showed that the expression of miR-1 together with miR-133b may control cell proliferation and cell cycle through c-Met protein expression modulation [47].

Another study conducted by Reid *et al.* showed that miR-1 can have a tumor-suppressor function in colorectal cancer by directly downregulating c-Met oncogene both at the RNA and protein levels and that reexpression of miR-1 leads to c-Met-driven reduction of cell proliferation and motility, identifying miR-1 downmodulation as one of the events that could enhance colorectal cancer progression [48].

Migliore’s group observed that miR-1 and miR-206 are highly expressed in skeletal muscle and investigated their role in the development of rhabdomyosarcoma. Interestingly, miR-1/206 expression levels were inversely correlated with c-Met, demonstrating that miR-1/206 suppressed c-Met expression in rhabdomyosarcoma and could function as a potent tumor suppressor in c-Met-over-expressing tumors [49].

### 2.6. Other miRNAs Regulating c-Met/HGF

Mariani and colleagues showed that miR-193a-5p was significantly over-expressed in patients undergoing neo-adjuvant chemotherapy (NACT) for ovarian cancer. They proved that patients who relapsed shortly after NACT exhibited the highest relative basal expression of both HGF and c-Met, indicating that mir-193a-5p, HGF and c-Met expression may help select patients that would benefit from these therapeutic regimens [50].

Another study conducted in HCC established that miR-26a exerted its antiangiogenesis function, at least in part, by inhibiting directly HGF and its downstream signaling pathway, in turn suppressing VEGFA production in HCC cells and impairing VEGFR2-signaling in endothelial cells [51].

The expression of miR-410 was inversely associated with c-Met in human glioma tissues. Thus, Chen demonstrated that miR-410 directly targeted c-Met in glioma cells and suggested they may function as a tumor suppressor in human gliomas [52].

In HCC cells, Buurman reported that miR-449 directly targets c-Met, leading to an increase of apoptosis and growth arrest of liver cancer cell lines. Expression of miR-449 slows growth of HCC xenograft tumors in mice, suggesting that this miR might function as a tumor suppressor [53].

MiR-198 directly targets c-Met via its 3' UTR and consequently its overexpression diminished HGF-induced phosphorylation of p44/42 MAPK in HCC cells, leading to an inhibition of cell migration and invasion in a c-Met-dependent manner [54].

A study conducted by Lee illustrated that miR-7515 plays an important role in the proliferation and migration of lung cancer cells through c-Met regulation. Indeed, miR-7515 downregulate in lung cancer compared with normal human lung cells and tissues and directly suppress c-Met, subsequently leading to decreased cell proliferation, migration and invasion in a lung cancer cell line [55].

Guo and colleagues showed that miR-101 over-expression decreased c-Met expression at both mRNA and protein levels inhibiting T24 cell lines migration and invasion [56,57].

**Figure 1:**
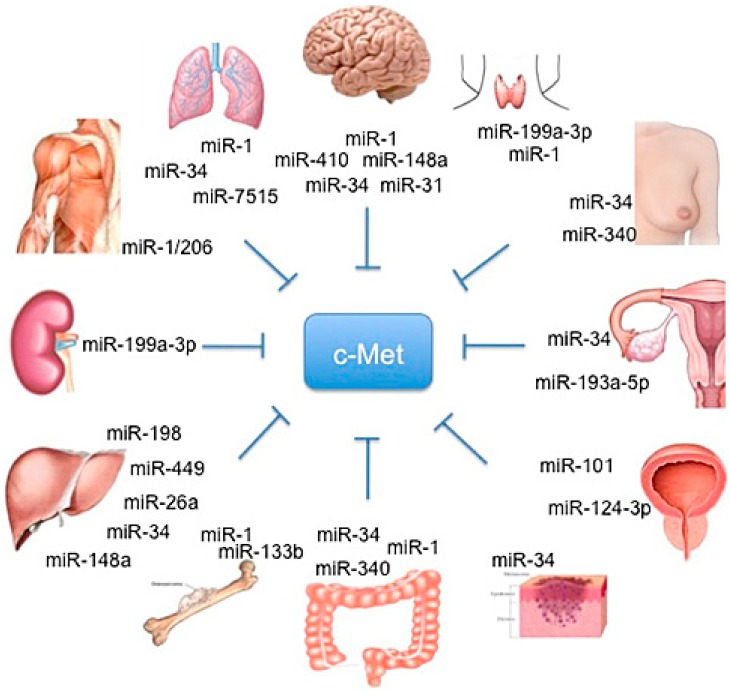
Schematic representation of organ-specific miR involved in HGF/c-MET regulation.

Korhan and colleagues found an inverse correlation between miR-181a-5p and c-Met expression in normal, cirrhotic and HCC liver tissues, and demostrated that miR-181a-5p is a direct target of c-Met. Furthemore, they showed that the knockdown of this miR leads to the activation of c-Met-mediated oncogenic signaling in hepatocarcinogenesis, thereby indicating that the reintroduction of tis miR may be a strategy to reduce c-Met activity [58] (Figure 1). Table 1 summarizes miRs regulating c-Met and other targets, the methods and the conditions by which the regulation was characterized.

## 3. Regulatory Circuits Controlled by c-Met and miRs

Important studies have pointed out that by regulating the expression of specific miRs, c-Met orchestrates the convergence of several EMT-associated pathways, including Dicer, SRC, PKC-ε and AKT, suggesting the possibility that c-Met targeting could be a strategy to control EMT and cancer progression.

Particularly, different studies conducted by Croce’s lab showed that c-Met is a direct regulator of miR expression.

In 2009, Garofalo *et al.*, showed that c-Met upregulates miR-221 and 222 expression through Jun transcriptional activation, which, in turn, confers resistance to TRAIL-induced cell death and increases tumorigenicity of lung and liver cancer cells by targeting PTEN and TIMP3 [59].

Previous studies reported that c-Met induces gefitinib resistance through persistent PI3K-AKT and ERK signaling activation [60].

In another paper, Garofalo *et al.* reported that c-Met expression downregulates miR-103 and miR-203, inducing gefitinib resistance, and epithelial−mesenchymal transition in non-small cells lung cancer (NSCLCs). They also showed that c-Met and EGF receptors induced the down-regulation of miR-30b and miR-30c expression [61].

The relationship between c-Met and miR-221/222 was confirmed by Acunzo *et al.*, which found that miR-130a overexpression reduced miR-221/222 levels in a c-Met-dependent manner. This finding highlighted how miR-130a, by targeting c-Met, was able to reduce miR-221/222 expression and, accordingly, TRAIL resistance in NSCLC cells. Thus, targeting c-Met and modulating miR-221/222 could be used not only to sensitize NSCLC to TRAIL-inducing apoptosis, but also in the prevention and inhibition of lung cancer [62].

The same group in 2013 demostrated that c-Met induces miR-23a-27a-24-2 expression. Furthermore, a member of this cluster, miR-27a, is able to downregulate c-Met and EGFR by either targeting directly their 3' UTRs or indirectly, by targeting Sprouty2. In summary, Acunzo *et al.* demonstrated a mechanism for c-Met regulation of EGFR expression in NSCLC that may give rise to further strategies for lung cancer treatment in the future [63].

**Figure 2:**
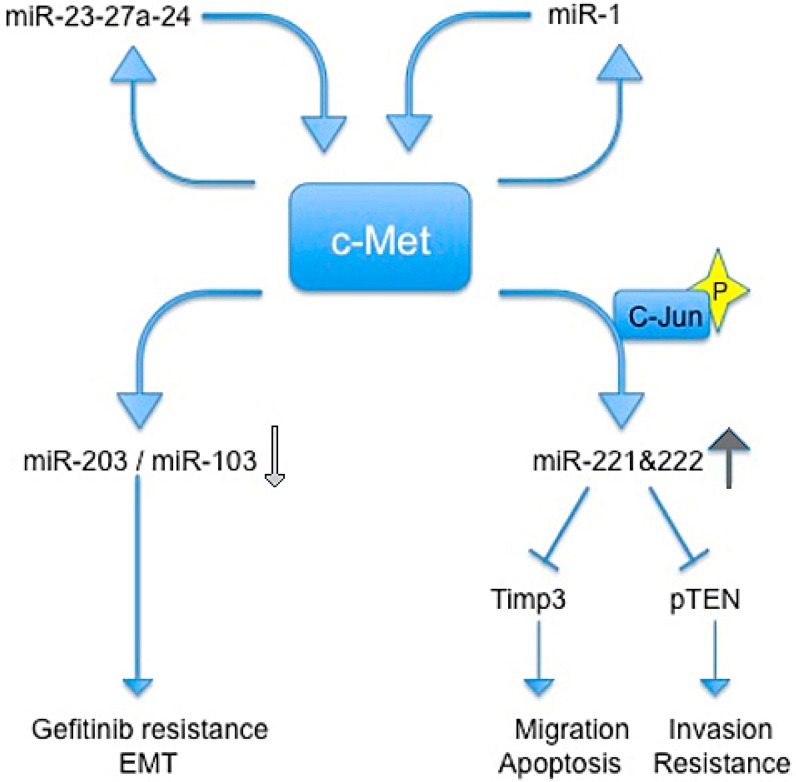
Schematic representation of regulatory circuits controlled by c-Met and miRNAs. Blue arrows indicate an induction of downstream signal trasduction, wherease blue shapes indicate a block of downstream signal trasduction. Light gray arrow indicates a reduction of miRs, the dark gray arrow indicates an increase of miRs.

Additionally, Migliore and colleagues identified a feedback loop between miR-1 and c-Met, resulting in their mutual regulation. They showed that concomitant downregulation of miR-1 and up-regulation of MACC1 leads to a c-Met induction and promotes cancer metastatis in colon cancer cells [49] (Figure 2).

**Table 1:** miRs targeting c-Met.

miRs	Identified Targets	Validation Methods	Disease or Conditions	References
miR-34	MET	Reporter assay, qRT-PCR, WB	U87, A172, LN-Z308, U373, T98G	[20]
	E2F3	Reporter assay and WB	Neuroblastoma	[15]
	CDKN1A	Reporter assay, qRT-PCR, WB Other	HEK293	[64]
miR-340-5p	MET	IHC, Reporter assay, qRT-PCR, WB, Other	primary breast cancer	[33]
miR-199a-3p	MET MAPK1	Reporter assay, WB, microarray	A549 cells	[31]
	MET	Reporter assay and WB	COS-7	[18]
miR-148a	MET	Reporter assay and WB	Hepatoma cells	[38]
miR-1	MET	Reporter assay and WB	A549	[44]
miR-193a-5p	MET	MicroArray	Ovarian cancer	[50]
	TP73	Reporter assay, qRT-PCR, WB	JHU-029, A549	[65]
miR-26a	HGF	Reporter assay	HCC	[51]
	PTEN	Reporter assay and WB	Thyroid cancer	[66]
miR-410	MET	Reporter assay and WB	Glioma cell lines	[52]
	MET	Reporter assay	HCC cell lines	[53]
	CDC25A	Reporter assay, qRT-PCR, WB, other	MCF10A, MCF7, Saos-2, SW480, U2OS-ER-E132, U2OS-ER-E2F1	[67]
miR-449	HDAC1	Reporter assay, qRTPCR, WB, other	prostate carcinoma cell lines	[68]
miR-198	MET	Reporter assay	HCC cell lines	[54]
miR-7515	MET	Reporter assay	Lung cancer cells and tissues	[55]
miR-101	MET	qRT-PCR and WB	Bladder cancer	[57]
	MYCN	Reporter assay and WB	HeLa	[69]
miR-181a-5p	MET	Reporter assay, qRT-PCR and WB	HCC	[58]

The abbreviations used are: miR, microRNA; qRT-PCR, quantitative real-time-PCR; WB, Western blot; Immunohistochemistry, IHC; HCC, Human hepatocellular carcinoma.

## 4. Conclusions and Outlook

Over-expression of c-Met contributes to the development a wide variety of human tumors. Counteracting the tumor-promoting activity of c-Met is therefore an attractive anticancer strategy that is currently being explored. Among different approaches targeting different levels of the c-Met expression, the characterization of the interplay between c-Met and miRs give another layer of targeting opportunities. Thus the rapidly growing understanding of the biology of miRs in cancer as well as the development in RNA-mediated therapeutic approaches in recent years, promises new and exciting modes of specific targeting in cancer.

In conclusion, further understanding of the effect of miRs on the c-Met expression will help to understand the pathogenesis of several types of cancer and provides a basis for novel targeted therapies for cancer treatment.

## References

[1] Cooper C.S., Park M., Blair D.G., Tainsky M.A., Huebner K., Croce C.M., vande Woude G.F. (1984). Molecular cloning of a new transforming gene from a chemically transformed human cell line. Nature.

[2] Giordano S., Ponzetto C., di Renzo M.F., Cooper C.S., Comoglio P.M. (1989). Tyrosine kinase receptor indistinguishable from the c-Met protein. Nature.

[3] Nakamura T., Nishizawa T., Hagiya M., Seki T., Shimonishi M., Sugimura A., Tashiro K., Shimizu S. (1989). Molecular cloning and expression of human hepatocyte growth factor. Nature.

[4] Bottaro D.P., Rubin J.S., Faletto D.L., Chan A.M., Kmiecik T.E., Vande Woude G.F., Aaronson S.A. (1991). Identification of the hepatocyte growth factor receptor as the *c-Met* proto-oncogene product. Science.

[5] Birchmeier C., Birchmeier W., Gherardi E., vande Woude G.F. (2003). Met, metastasis, motility and more. Nat. Rev. Mol. Cell Biol..

[6] Lai A.Z., Abella J.V., Park M. (2009). Crosstalk in Met receptor oncogenesis. Trends Cell Biol..

[7] Kroll J., Loedige I., Filipowicz W. (2010). The widespread regulation of microRNA biogenesis, function and decay. Nat. Rev. Genet..

[8] Ameres S.L., Zamore P.D. (2013). Zamore Diversifying microRNA sequence and function. Nat. Rev. Mol. Cell Biol..

[9] Esquela-Kerscher A., Slack F.J. (2006). Oncomirs—MicroRNAs with a role in cancer. Nat. Rev. Cancer.

[10] Lu J., Getz G., Miska E.A., Alvarez-Saavedra E., Lamb J., Peck D., Sweet-Cordero A., Ebert B.L., Mak R.H., Ferrando A.A. (2005). MicroRNA expression profiles classify human cancers. Nature.

[11] Calin G.A., Ferracin M., Cimmino A., di Leva G., Shimizu M., Wojcik S.E., Iorio M.V., Visone R., Sever N.I., Fabbri M. (2006). A microRNA signature associated with prognosis and progression in chronic lymphocytic leukemia. N. Engl. J. Med..

[12] Volinia S., Calin G.A., Liu C.G., Ambs S., Cimmino A., Petrocca F., Visone R., Iorio M., Roldo C., Ferracin M. (2006). A microRNA expression signature of human solid tumors defines cancer gene targets. Proc. Natl. Acad. Sci. USA.

[13] Agostini M., Knight R.A. (2014). miR-34: From bench to bedside. Oncotarget.

[14] Bagchi A., Mills A.A. (2008). The Quest for the 1p36 Tumor Suppressor. Cancer Res..

[15] Welch C., Chen Y., Stallings R.L. (2007). MicroRNA-34a functions as a potential tumor suppressor by inducing apoptosis in neuroblastoma cells. Oncogene.

[16] Vogt M., Munding J., Grüner M., Liffers S.-T., Verdoodt B., Hauk J., Steinstraesser L., Tannapfel A., Hermeking H. (2011). Frequent concomitant inactivation of miR-34a and miR-34b/c by CpG methylation in colorectal, pancreatic, mammary, ovarian, urothelial, and renal cell carcinomas and soft tissue sarcomas. Virchows Arch..

[17] He L., He X., Lim L.P., de Stanchina E., Xuan Z., Liang Y., Xue W., Zender L., Magnus J., Ridzon D. (2007). A microRNA component of the p53 tumour suppressor network. Nature.

[18] Migliore C., Petrelli A., Ghiso E., Corso S., Capparuccia L., Eramo A., Comoglio P.M., Giordano S. (2008). MicroRNAs impair MET-mediated invasive growth. Cancer Res..

[19] Corney D.C., Hwang C.I., Matoso A., Vogt M., Flesken-Nikitin A., Godwin A.K., Kamat A.A., Sood A.K., Ellenson L.H., Hermeking H. (2010). Frequent downregulation of miR-34 family in human ovarian cancers. Clin. Cancer Res..

[20] Li Y., Guessous F., Zhang Y., Dipierro C., Kefas B., Johnson E., Guessous F., Zhang Y., Dipierro C., Kefas B. (2009). MicroRNA-34a inhibits glioblastoma growth by targeting multiple oncogenes. Cancer Res..

[21] Guessous F., Zhang Y., Kofman A., Catania A., Li Y., Schiff D., Purow B., Abounader R. (2010). microRNA-34a is tumor suppressive in brain tumors and glioma stem cells. Cell Cycle.

[22] Dang Y., Luo D., Rong M., Chen G. (2013). Underexpression of miR-34a in hepatocellular carcinoma and its contribution towards enhancement of proliferating inhibitory effects of agents targeting c-MET. PLoS One.

[23] Cai K.M., Bao X.L., Kong X.H., Jinag W., Mao M.R., Chu J.S., Huang Y.J., Zhao X.J. (2010). Hsa-miR-34c suppresses growth and invasion of human laryngeal carcinoma cells via targeting c-Met. Int. J. Mol. Med..

[24] Zhou J.Y., Chen X., Zhao J., Bao Z., Chen X., Zhang P., Liu Z.F., Zhou J.Y. (2014). MicroRNA-34a overcomes HGF-mediated gefitinib resistance in EGFR mutant lung cancer cells partly by targeting MET. Cancer Lett..

[25] Fornari F., Milazzo M., Chieco P., Negrini M., Calin G.A., Grazi G.L., Pollutri D., Croce C.M., Bolondi L., Gramantieri L. (2010). MiR-199a-3p regulates mTOR and c-Met to influence the doxorubicin sensitivity of human hepatocarcinoma cells. Cancer Res..

[26] Gramantieri L., Ferracin M., Fornari F., Veronese A., Sabbioni S., Liu C.G., Calin G.A., Giovannini C., Ferrazzi E., Grazi G.L. (2007). Cyclin G1 is a target of miR-122a, a microRNA frequently down-regulated in human hepatocellular carcinoma. Cancer Res..

[27] Ichimi T., Enokida H., Okuno Y., Kunimoto R., Chiyomaru T., Kawamoto K., Kawahara K., Toki K., Kawakami K., Nishiyama K. (2009). Identification of novel microRNA targets based on microRNA signatures in bladder cancer. Int. J. Cancer.

[28] Iorio M.V., Visone R., di Leva G., Donati V., Petrocca F., Casalini P., Taccioli C., Volinia S., Liu C.G., Alder H. (2007). MicroRNA signatures in human ovarian cancer. Cancer Res..

[29] Huang J., Dong B., Zhang J., Kong W., Chen Y., Xue W., Liu D., Huang Y. (2014). miR-199a-3p inhibits hepatocyte growth factor/c-Met signaling in renal cancer carcinoma. Tumour Biol..

[30] Landgraf P., Rusu M., Sheridan R., Sewer A., Iovino N., Aravin A., Pfeffer S., Rice A., Kamphorst A.O., Landthaler M. (2007). A mammalian microRNA expression atlas based on small RNA library sequencing. Cell.

[31] Kim S., Lee U.J., Kim M.N., Lee E.J., Kim J.Y., Lee M.Y., Choung S., Kim Y.J., Choi Y.C. (2008). MicroRNA miR-199a* regulates the *MET* proto-oncogene and the downstream extracellular signal-regulated kinase 2 (ERK2). J. Biol. Chem..

[32] Minna E., Romeo P., de Cecco L., Dugo M., Cassinelli G., Pilotti S., Degl’Innocenti D., Lanzi C., Casalini P., Pierotti M.A. (2014). miR-199a-3p displays tumor suppressor functions in papillary thyroid carcinoma. Oncotarget.

[33] Wu Z.S., Wu Q., Wang C.Q., Wang X.N., Huang J., Zhao J.J., Mao S.S., Zhang G.H., Xu X.C., Zhang N. (2011). miR-340 inhibition of breast cancer cell migration and invasion through targeting of oncoprotein c-Met. Cancer.

[34] Sun Y., Zhao X., Zhou Y., Hu Y. (2012). miR-124, miR-137 and miR-340 regulate colorectal cancer growth via inhibition of the Warburg effect. Oncol. Rep..

[35] Takeyama H., Yamamoto H., Yamashita S., Wu X., Takahashi H., Nishimura J., Haraguchi N., Miyake Y., Suzuki R., Murata K. (2014). Decreased miR-340 expression in bone marrow is associated with liver metastasis of colorectal cancer. Mol. Cancer Ther..

[36] Liffers S.T., Munding J.B., Vogt M., Kuhlmann J.D., Verdoodt B., Nambiar S., Maghnouj A., Mirmohammadsadegh A., Hahn S.A., Tannapfel A. (2011). MicroRNA-148a is down-regulated in human pancreatic ductal adenocarcinomas and regulates cell survival by targeting CDC25B. Lab. Investig..

[37] Fujita Y., Kojima K., Ohhashi R., Hamada N., Nozawa Y., Kitamoto A., Sato A., Kondo S., Kojima T., Deguchi T. (2010). MiR-148a attenuates paclitaxel resistance of hormone-refractory, drug-resistant prostate cancer PC3 cells by regulating MSK1 expression. J. Biol. Chem..

[38] Zhang H., Li Y., Huang Q., Ren X., Hu H., Sheng H., Lai M. (2011). MiR-148a promotes apoptosis by targeting Bcl-2 in colorectal cancer. Cell Death Differ..

[39] Yu J., Li Q., Xu Q., Liu L., Jiang B. (2011). MiR-148a inhibits angiogenesis by targeting ERBB3. J. Biomed. Res..

[40] Zheng B., Liang L., Wang C., Huang S., Cao X., Zha R., Liu L., Jia D., Tian Q., Wu J. (2011). MicroRNA-148a suppresses tumor cell invasion and metastasis by downregulating ROCK1 in gastric cancer. Clin. Cancer Res..

[41] Zhang J.P., Zeng C., Xu L., Gong J., Fang J.H., Zhuang S.M. (2014). MicroRNA-148a suppresses the epithelial-mesenchymal transition and metastasis of hepatoma cells by targeting Met/Snail signaling. Oncogene.

[42] Xu Q., Jiang Y., Yin Y., Li Q., He J., Jing Y., Qi Y.T., Xu Q., Li W., Lu B. (2013). A regulatory circuit of miR-148a/152 and DNMT1 in modulating cell transformation and tumor angiogenesis through IGF-IR and IRS1. J. Mol. Cell Biol..

[43] Gailhouste L., Gomez-Santos L., Hagiwara K., Hatada I., Kitagawa N., Kawaharada K., Thirion M., Kosaka N., Takahashi R.U., Shibata T. (2013). miR-148a plays a pivotal role in the liver by promoting the hepatospecific phenotype and suppressing the invasiveness of transformed cells. Hepatology.

[44] Nasser M.W., Datta J., Nuovo G., Kutay H., Motiwala T., Majumder S., Wang B., Suster S., Jacob S.T., Ghoshal K. (2008). Down-regulation of micro-RNA-1 (miR-1) in lung cancer. Suppression of tumorigenic property of lung cancer cells and their sensitization to doxorubicin-induced apoptosis by miR-1. J. Biol. Chem..

[45] Yip L., Kelly L., Shuai Y., Armstrong M.J., Nikiforov Y.E., Carty S.E., Nikiforova M.N. (2011). MicroRNA signature distinguishes the degree of aggressiveness of papillary thyroid carcinoma. Ann. Surg. Oncol..

[46] Duan Z., Choy E., Harmon D., Liu X., Susa M., Mankin H., Hornicek F. (2011). MicroRNA-199a-3p is downregulated in human osteosarcoma and regulates cell proliferation and migration. Mol. Cancer Ther..

[47] Novello C., Pazzaglia L., Cingolani C., Conti A., Quattrini I., Manara M.C., Tognon M., Picci P., Benassi M.S. (2013). miRNA expression profile in human osteosarcoma: Role of miR-1 and miR-133b in proliferation and cell cycle control. Int. J. Oncol..

[48] Reid J.F., Sokolova V., Zoni E., Lampis A., Pizzamiglio S., Bertan C., Zanutto S., Perrone F., Camerini T., Gallino G. (2012). miRNA profiling in colorectal cancer highlights miR-1 involvement in MET-dependent proliferation. Mol. Cancer Res..

[49] Migliore C., Martin V., Leoni V.P., Restivo A., Atzori L., Petrelli A., Isella C., Zorcolo L., Sarotto I., Casula G. (2012). MiR-1 downregulation cooperates with MACC1 in promoting C-MET overexpression in human colon cancer. Clin. Cancer Res..

[50] Mariani M., McHugh M., Petrillo M., Sieber S., He S., Andreoli M., Wu Z., Fiedler P., Scambia G., Shahabi S. (2014). HGF/c-Met axis drives cancer aggressiveness in the neo-adjuvant setting of ovarian cancer. Oncotarget.

[51] Yang X., Zhang X.F., Lu X., Jia H.L., Liang L., Dong Q.Z., Ye Q.H., Qin L.X. (2014). MicroRNA-26a suppresses angiogenesis in human hepatocellular carcinoma by targeting hepatocyte growth factor–c-Met pathway. Hepatology.

[52] Chen L., Zhang J., Feng Y., Li R., Sun X., Du W., Piao X., Wang H., Yang D., Sun Y. (2012). MiR-410 regulates MET to influence the proliferation and invasion of glioma. Int. J .Biochem. Cell Biol..

[53] Buurman R., Gürlevik E., Schäffer V., Eilers M., Sandbothe M., Kreipe H., Wilkens L., Schlegelberger B., Kühnel F., Skawran B. (2012). Histone deacetylases activate hepatocyte growth factor signaling by repressing microRNA-449 in hepatocellular carcinoma cells. Gastroenterology.

[54] Tan S., Li R., Ding K., Lobie P.E., Zhu T. (2011). miR-198 inhibits migration and invasion of hepatocellular carcinoma cells by targeting the HGF/c-MET pathway. FEBS Lett..

[55] Lee J.M., Yoo J.K., Yoo H., Jung H.Y., Lee D.R., Jeong H.C., Oh S.H., Chung H.M., Kim J.K. (2013). The novel miR-7515 decreases the proliferation and migration of human lung cancer cells by targeting c-Met. Mol. Cancer Res..

[56] Guo F., Parker Kerrigan B.C., Yang D., Hu L., Shmulevich I., Sood A.K., Xue F., Zhang W. (2014). Post-transcriptional regulatory network of epithelial-to-mesenchymal and mesenchymal-to-epithelial transitions. J. Hematol. Oncol..

[57] Hu Z., Lin Y., Chen H., Mao Y., Wu J., Zhu Y., Xu X., Xu X., Li S., Zheng X. (2013). MicroRNA-101 suppresses motility of bladder cancer cells by targeting c-Met. Biochem. Biophys. Res. Commun..

[58] Korhan P., Erdal E., Atabey N. (2014). MiR-181a-5p is downregulated in hepatocellular carcinoma and suppresses motility, invasion and branching-morphogenesis by directly targeting c-Met. Biochem. Biophys. Res. Commun..

[59] Garofalo M., di Leva G., Romano G., Nuovo G., Suh S.S., Ngankeu A., Taccioli C., Pichiorri F., Alder H., Secchiero P. (2009). miR-221&miR-222 regulate TRAIL resistance and enhance tumorigenicity through PTEN and TIMP3 downregulation. Cancer Cell..

[60] Li H., Zhou S., Li X., Wang D., Wang Y., Zhou C., Schmid-Bindert G. (2013). Gefitinib-resistance is related to BIM expression in non-small cell lung cancer cell lines. Cancer Biother. Radiopharm..

[61] Garofalo M., Romano G., di Leva G., Nuovo G., Jeon Y.J., Ngankeu A., Sun J., Lovat F., Alder H., Condorelli G. (2011). EGFR and MET receptor tyrosine kinase-altered microRNA expression induces tumorigenesis and gefitinib resistance in lung cancers. Nat. Med..

[62] Acunzo M., Visone R., Romano G., Veronese A., Lovat F., Palmieri D., Bottoni A., Garofalo M., Gasparini P., Condorelli G. (2012). miR-130a targets MET and induces TRAIL-sensitivity in NSCLC by downregulating miR-221 and 222. Oncogene.

[63] Acunzo M., Romano G., Palmieri D., Laganá A., Garofalo M., Balatti V., Drusco A., Chiariello M., Nana-Sinkam P., Croce C.M. (2013). Cross-talk between MET and EGFR in non-small cell lung cancer involves miR-27a and Sprouty2. Proc. Natl. Acad. Sci. USA.

[64] Wu S., Huang S., Ding J., Zhao Y., Liang L., Liu T., Zhan R., He X. (2010). Multiple microRNAs modulate p21Cip1/Waf1 expression by directly targeting its 3' untranslated region. Oncogene.

[65] Ory B., Ramsey M.R., Wilson C., Vadysirisack D.D., Forster N., Rocco J.W., Rothenberg S.M., Ellisen L.W. (2011). A microRNA-dependent program controls p53-independent survival and chemosensitivity in human and murine squamous cell carcinoma. J. Clin. Invest..

[66] Visone R., Pallante P., Vecchione A., Cirombella R., Ferracin M., Ferraro A., Volinia S., Coluzzi S., Leone V., Borbone E. (2007). Specific microRNAs are downregulated in human thyroid anaplastic carcinomas. Oncogene.

[67] Yang X., Feng M., Jiang X., Wu Z., Li Z., Aau M., Yu Q. (2009). miR-449a and miR-449b are direct transcriptional targets of E2F1 and negatively regulate pRb-E2F1 activity through a feedback loop by targeting CDK6 and CDC25A. Genes Dev..

[68] Noonan E.J., Place R.F., Pookot D., Basak S., Whitson J.M., Hirata H., Giardina C., Dahiya R. (2009). miR-449a targets HDAC-1 and induces growth arrest in prostate cancer. Oncogene.

[69] Lewis B.P., Shih I.H., Jones-Rhoades M.W., Bartel D.P., Burge C.B. (2003). Prediction of mammalian microRNA targets. Cell.

